# Acute risk for hepatitis E virus infection among HIV-1-positive pregnant women in central Africa

**DOI:** 10.1186/1743-422X-9-254

**Published:** 2012-10-31

**Authors:** Mélanie Caron, Julie Bouscaillou, Mirdad Kazanji

**Affiliations:** 1 Unité de Rétrovirologie, Centre International de Recherches Médicales, Franceville, BP, 769, Gabon; 2 Institut Pasteur de Bangui, Réseau International des Instituts Pasteur, Bangui, Central African Republic

**Keywords:** HEV prevalence, HIV-1 and HTLV-1 infections, Pregnant women, Gabon, Central Africa

## Abstract

**Background:**

Hepatitis E virus (HEV), an enterically transmitted pathogen, is highly endemic in several African countries. Pregnant women are at particularly high risk for acute or severe hepatitis E. In Gabon, a central African country, the prevalence of antibodies to HEV among pregnant women is 14.1%. Recent studies have demonstrated unusual patterns of hepatitis E (chronic hepatitis, cirrhosis) among immunodeficient patients.

**Findings:**

We investigated the prevalence of antibodies to HEV among pregnant women infected with HIV-1 or HTLV-1 in Gabon. Of 243 samples collected, 183 were positive for HIV-1 and 60 for HTLV-1; 16 women (6.6%) had IgG antibodies to HEV. The seroprevalence was higher among HIV-1-infected women (7.1%) than HTLV-1-infected women (5.0%). Moreover, the HIV-1 viral load was significantly increased (*p* ≤ 0.02) among women with past-HEV exposure (1.3E+05 *vs* 5.7E+04 copies per ml), whereas no difference was found in HTLV-1 proviral load (9.0E+01 *vs* 1.1E+03 copies per ml).

**Conclusions:**

These data provide evidence that HIV-1-infected women are at risk for acute or severe infection if they are exposed to HEV during pregnancy, with an increased viral load.

## Findings

Hepatitis E virus (HEV) is an enterically transmitted pathogen that causes widescale epidemics of acute hepatitis in highly HEV-endemic areas such as Africa, Asia and the Middle East [1]. Sporadic cases are also observed in Europe, Japan, Russia and the USA, where HEV is spread mainly by zoonotic foodborne transmission [2]. Although perinatal and bloodborne transmission may occur, the main route of HEV transmission worldwide remains ingestion of fecal-contaminated water [3].

Many HEV outbreaks have been described in Africa, such as that recently observed in Uganda, with over 10 000 cases of acute hepatitis and 160 deaths [4]. The prevalence of antibodies to HEV varies widely in Africa, from 4.4% in the rural population of Ghana to 84.3% among pregnant women in Egypt [5,6]. In a previous study, we found a prevalence of 14.1% among pregnant women in Gabon [7].

HEV is responsible for self-limiting or acute hepatitis, the severity ranging from benign to fulminant forms [8]. Hepatitis E is associated with a mortality rate of ≤ 4%, in particular among young adults, and up to 20% among pregnant women [9]. Obstetric complications due to HEV infection might be partly explained by hormonal changes and immune factors [10]. A study on maternal and fetal outcomes in India showed that pregnant women with acute hepatitis E had a 2.7 times higher relative risk for fulminant hepatic failure and a 6.0 times higher risk for mortality [11].

Persistent carriage of HEV has been described recently, with cases of chronic hepatitis and cirrhosis among organ-transplant recipients under immunosuppressive therapy [12-14]. In studies of acute hepatitis E among HIV-1-infected patients [15,16], it has been suggested that hepatitis E can become chronic in people with severe immunodeficiency [17]. Long-term carriage of HEV might therefore warrant increased awareness and vigilance in cases of HIV-1 infection.

Previous studies conducted by our group among pregnant women in Gabon showed that the prevalence of HIV-1 infection is 6.3% and that of another human retrovirus, HTLV-1, is 2.1% [18,19]. The two viruses (HIV-1 and HTLV-1) have identical modes of transmission (sexual) and induce immunological disorders. As co-infections might occur in populations that are highly exposed to common risk factors and co-infection of a woman with a pre-existing infection with HIV-1 or HTLV-1 might increase the severity of disease, we investigated the prevalence of antibodies to HEV among HIV-1- and HTLV-1-infected pregnant women in Gabon. A cohort of 243 pregnant women was followed at the International Centre for Medical Research in Franceville, Gabon. Blood samples were collected anonymously, and only sociodemographic data were retained after informed consent. The study obtained ethical clearance from the Gabonese public health authorities (Ministry of Health) and from the Gabonese scientific and national ethical committees (Authorization no 093/MSP/SG/SGAQM).

After serological and confirmation assays for diagnosis of HIV-1 and HTLV-1/2, the HIV-1 viral load was determined with “Generic HIV Charge Viral (Biocentric, France)” and the HTLV-1 proviral load as previously described by Besson et al. [20]. IgG antibodies to HEV were measured with an ELISA (TMB) Kit (Genelabs Diagnostics, Singapore) according to the manufacturer’s instructions. None of pregnant women in our study was co-infected with HIV-2, HTLV-2, HBV/HDV or HCV. Statistical analyses were conducted with STATA 11.0 software.

Of the 243 pregnant women, 183 were infected with HIV-1 and 60 with HTLV-1 (Table 1). The mean age of the study population was 28.2 ± 6.5 years (range, 14–43 years). The mean age of the HIV-1-infected women was 27.9 ± 6.2 years (range, 15–43 years), and the median viral load was 6.1E+04 copies per ml (first quartile, 1.5E+04 copies per ml; third quartile, 2.6E+05 copies per ml). The mean age of the HTLV-1-infected women was 29.1 ± 7.1 years (range, 16–42 years), and the median proviral load was 9.3E+02 copies per ml (first quartile, 2.7E+02 copies per ml; third quartile, 3.8E+03 copies per ml).

**Table 1:** Viral status by prevalence of antibodies to hepatitis E virus (HEV) among pregnant women in Gabon, central Africa

**Viral status**	**HEV status**	**No. (%)**	**Mean age (years) (SD)**	***p***	**Median viral load (copies/ml) [FQ-TQ]**	***p***
HIV-1+	HEV+	13	26.3 (4.5)	NS	1.3E+05 [5.1E+04-9.2E+05]	**≤ 0.02**
	HEV–	170	28.0 (6.3)		5.7E+04 [1.5E+04-2.4E+05]	
	**Total**	**13/183 (7.1)**	**27.9 (6.2)**		**6.1E+04 [1.5E+04-2.6E+05]**	
HTLV-1+	HEV+	3	33.7 (5.4)	NS	9.0E+01 [6.0E+00-5.7E+02]	NS
	HEV–	57	28.8 (7.1)		1.1E+03 [2.8E+02-3.9E+03]	
	**Total**	**3/60 (5.0)**	**29.1 (7.1)**		**9.3E+02 [2.7E+02-3.8E+03]**	
Total	HEV+	16	27.8 (5.5)	NS	–	-
	HEV–	227	28.2 (6.5)		–	
	**Total**	**16/243 (6.6)**	**28.2 (6.5)**		*–*	

HEV+: IgG antibodies to HEV in serum; HIV-1, human immunodeficiency virus type 1; HTLV-1, human T-cell leukemia virus type 1; No., number of pregnant women; SD, standard deviation; [FQ–TQ], first quartile–third quartile; NS, not significant; –, not done.

Significance level (*p* < 0.05) calculated with Student *t* test for age, Fisher exact test for viral status, and Wilcoxon-Mann–Whitney test for viral load, as the distribution was not normal.

**Figure 1:**
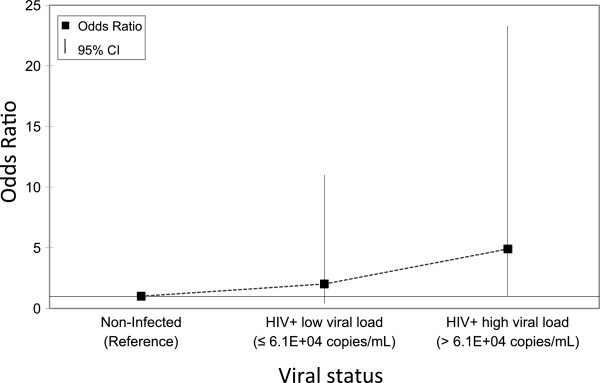
Odds ratios for having IgG antibodies to hepatitis E virus (HEV) by human immunodeficiency virus (HIV-1) status and viral load.

Sixteen of the 243 pregnant women (6.6%) had IgG antibodies to HEV (Table 1). The seroprevalence did not depend significantly on viral status (HIV-1 positive or HTLV-1-positive) or age. HTLV-1-infected women with past exposure to HEV had lower proviral loads than those without past exposure. Nevertheless, the sample size and seroprevalence in this population were low, making it difficult to detect a significant difference. Conversely, the seroprevalence tended to increase with HIV-1 viral load (*p* ≤ 0.02 with the Cuzick non-parametric test for trend [21]), when HIV-1 status was stratified into three groups: uninfected and HIV-1 positive, HIV-1 positive with viral load ≤ median and HIV-1 positive, HIV-1 positive with viral load > median and HIV-1 positive (Table 1, Figure 1). HIV-1-infected women with a high viral load were at higher risk for acute or severe hepatitis E.

Previously, we found HEV prevalences of 6.4% in rural and 13.5% in urban Gabonese pregnant women, which were significantly different (*p* < 0.05) [7]. In this study, we found that the risk for HEV infection of pregnant women infected with HIV-1 or HTLV-1 was similar to that of women living in rural areas. This finding corroborates our previous report of endemic HEV circulation in Gabon and indicates active autochthonous HEV transmission among women of reproductive age. While no effect of past HEV exposure was found on the prevalence of HTLV-1 infection, an increased prevalence of antibodies to HEV was associated with a high HIV-1 load.

It is possible that HIV-1 infection predisposes to HEV acquisition, as suggested in a study in the Russian Federation, which showed an association between a higher HEV prevalence and more advanced HIV-1 related disease [22]. Recently, persistent carriage of HEV has been observed among patients with HIV-1 infection. This might usually be overlooked because of common drug-induced liver injury among patients receiving antiretroviral therapy [23]. HEV infection could, however, represent a differential diagnosis of hepatitis in pregnancy [24]. As in our study, most HIV-1-infected pregnant women do not have HEV antibodies, placing them at increased risk for acute or severe hepatitis E in an area endemic for both viruses.

HIV-1-infected pregnant women in Gabon appear to have a specific risk for HEV acquisition, with an increased viral load. No studies of hepatitis E have been conducted in the general population of Gabon, and the sources of infection remain unknown. In conclusion, HEV might be an important unrecognized cause of fatal hepatitis, particularly among HIV-1-positive pregnant women.

## Competing interests

The authors declare that they have no competing interests.

## Authors’ contributions

MC carried out the serological and molecular studies, JB performed the statistical analysis. MC, JB and MK conceived and designed the study and were involved in drafting the manuscript. All the authors read and approved the final manuscript.
